# Development of a Protein Microarray Chip with Enhanced Fluorescence for Identification of Semen and Vaginal Fluid

**DOI:** 10.3390/s18113874

**Published:** 2018-11-10

**Authors:** Naseem Abbas, Xun Lu, Mohsin Ali Badshah, Jung Bin In, Won Il Heo, Kui Young Park, Mi-Kyung Lee, Cho Hee Kim, Pilwon Kang, Woo-Jin Chang, Seok-Min Kim, Seong Jun Seo

**Affiliations:** 1 Department of Mechanical Engineering, Chung-Ang University, 84 Heukseok-ro, Dongjak-gu, Seoul 06974, Korea; naseem@cau.ac.kr (N.A.); luxun@cau.ac.kr (X.L.); mohsinali@cau.ac.kr (M.A.B.); jbin@cau.ac.kr (J.B.I.); 2 Department of Dermatology, Chung-Ang University Hospital, 102 Heukseok-ro, Dongjak-gu, Seoul 06973, Korea; one1x@cau.ac.kr (W.I.H.); kyky@caumc.or.kr (K.Y.P.); 3 Department of Laboratory Medicine, Chung-Ang University Hospital, 102 Heukseok-ro, Dongjak-gu, Seoul 06973, Korea; cpworld@cau.ac.kr; 4 Forensic DNA Division, National Forensic Service, 10 Ipchun-ro, Wonju-si, Gangwon-do 26460, Korea; chkim1220@korea.kr (C.H.K); kpwon7@korea.kr (P.K); 5 Department of Mechanical Engineering, University of Wisconsin-Milwaukee, 3200 N Cramer St, Milwaukee, WI 53211, USA; wjchang@uwm.edu

**Keywords:** metal-enhanced fluorescence, body fluid identification, glancing angle deposition, microarray analysis, Ag nanorod, semen, vaginal fluid

## Abstract

The detection of body fluids has been used to identify a suspect and build a criminal case. As the amount of evidence collected at a crime site is limited, a multiplex identification system for body fluids using a small amount of sample is required. In this study, we proposed a multiplex detection platform using an Ag vertical nanorod metal enhanced fluorescence (MEF) substrate for semen and vaginal fluid (VF), which are important evidence in cases of sexual crime. The Ag nanorod MEF substrate with a length of 500 nm was fabricated by glancing angle deposition, and amino functionalization was conducted to improve binding ability. The effect of incubation time was analyzed, and an incubation time of 60 min was selected, at which the fluorescence signal was saturated. To assess the performance of the developed identification chip, the identification of semen and VF was carried out. The developed sensor could selectively identify semen and VF without any cross-reactivity. The limit of detection of the fabricated microarray chip was 10 times better than the commercially available rapid stain identification (RSID) Semen kit.

## 1. Introduction

The identification of body fluids plays a vital role in the criminal justice system by providing scientifically based information through the analysis of physical evidence. Among the various body fluids, semen [1,2] and vaginal fluid (VF) [3,4] are important in cases of sexual crime. Although epigenetic markers have been used for the identification of semen and VF [5], this method requires complex sample treatment for identification. Protein biomarkers have also been used to detect semen and VF, which do not need complex treatment. Acid phosphatase [6], semenogelin 1 and 2 [7], and prostate-specific antigen [8] have been used as protein biomarkers for human semen. In addition, various methods have been developed for the identification of human semen using protein biomarkers, such as electrophoresis [6], immunochromatography [7,9], and Raman spectroscopy [8,10]. For the identification of VF, 17 beta-estradiol [11,12], human small proline-rich protein 3 [13], human fatty acid-binding protein 5 [13], and cervical protein [14] have been used as biomarkers, and the electrochemical enzyme-linked immunosorbent assay [11,12] and differential pulse voltammetry [11,15] methods have been used. Although some identification methods using protein biomarkers are commercialized as a pre-screening tool for forensic analysis [9,16], previous methods require a relatively large amount of sample (generally 1.5~2.0 mL) [9,16] to detect a single body fluid. Considering that the amount of sample collected from a crime site is limited and that the remaining sample can be used for other analyses, sample waste should be minimized in the pre-screening process. A protein microarray biosensor platform may be an ideal solution for the identification of multiple body fluids with a small amount of sample waste [17,18]. Fluorescence-based detection technology has been widely used for analysis in microarray biosensors. The application of metal-enhanced fluorescence (MEF) is regarded as a powerful approach for improving the sensitivity of a microarray biosensor and reducing the sample amount required for analysis, in which fluorophores interact with metallic nanostructures to enhance fluorescence [19,20]. Various fabrication techniques for MEF substrates have been reported in the literature, such as electron beam lithography [21], solution-seeded nanorod synthesis [22], wet chemistry [23], electrochemistry [24,25], and glancing angle deposition (GLAD) [26,27,28,29]. Among the methods for fabricating MEF substrates, GLAD can be considered to be a suitable method for fabricating large-area MEF substrates at a low cost with high uniformity [29]. In this study, we proposed a highly sensitive semen and VF identification (HSV-ID) chip using GLAD-fabricated Ag nanorods as the MEF substrate. The GLAD Ag nanorods substrate with a nanorod length of 500 nm was fabricated, and a chemical treatment process was performed to increase the immobilization efficiency between the MEF substrate and the antibodies for semen and VF. After spotting the antibodies on the substrate, the blocking, washing, and detection protocols were developed. To evaluate the developed HSV-ID chip, the detection limit of the HSV-ID chip was analyzed and compared with that of the commercial RSID™-Semen kit [9], and the selectivity for semen and VF was also analyzed.

## 2. Fabrication of the GLAD MEF Substrate

**Figure 1:**
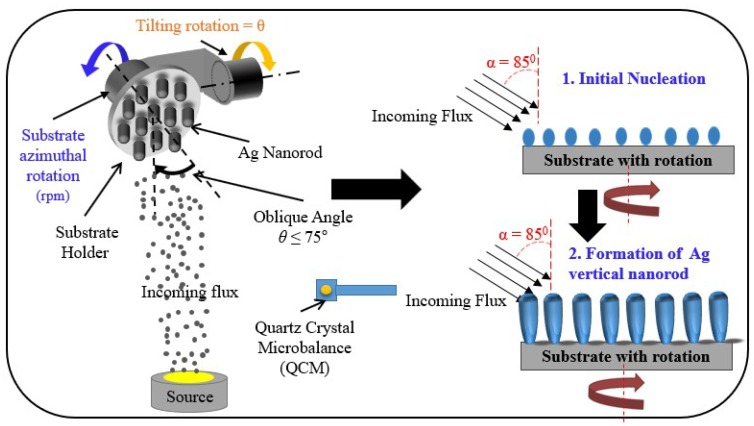
Schematic of the glancing angle deposition (GLAD) system with vertical Ag nanorod formation.

A MEF substrate for the HSV-ID chip was fabricated by GLAD of Ag nanorods on a slide glass substrate. GLAD is a well-known single step physical vapor deposition technique in which the evaporation flux impinges above the rotating substrate at an incident oblique angle of ≥75° [30], resulting in a highly porous film of isolated nanorods. Figure 1 shows the schematic of the GLAD system with vertical Ag nanorod formation. The evaporated nanoparticles from the source were randomly distributed on the substrate early in the GLAD process. Since subsequent particles from the source cannot reach the shadow areas of the initially distributed nanoparticles, the initial particles grew as the nanorods tilted towards the incoming material. When the substrate was rotating during deposition, the growing direction of the tilted nanorods gradually changed. A spiral nanorod can be obtained at a slow rotation speed, and a vertical nanorod can be obtained at a fast rotation speed. The nanorod diameter was determined by the atomic mobility of the material, which may be affected by the material properties, vacuum conditions, and temperature. The nanorod density was determined by the incident angle of the evaporation flux, and the length was determined by the evaporation time.

**Figure 2:**
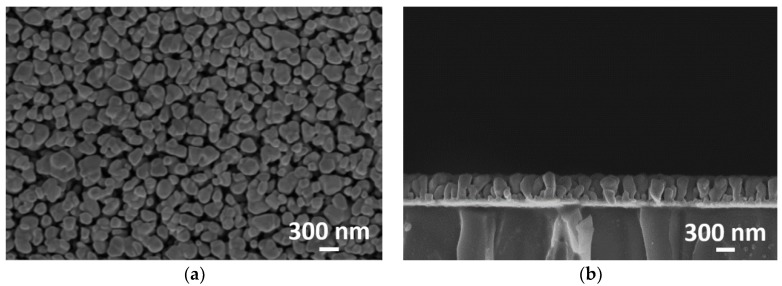
(**a**) Top-view and (**b**) cross-sectional view of the scanning electron microscopy images of the fabricated Ag vertical nanorods metal-enhanced fluorescence (MEF) substrate with a nanorod length of 500 nm.

Vertical Ag nanorods were fabricated on a conventional slide glass substrate with a size of 75 × 25 mm^2^ by GLAD. Prior to mounting the substrate on the jig of the electron beam evaporation system, the substrate was cleaned with acetone, isopropyl alcohol, and de-ionized (DI) water. For microarray biochip applications, which require several washing steps, good adhesion is necessary between the substrate and Ag nanorods. A Ni layer (10 nm thickness) and an Ag layer (50 nm thickness) were sequentially deposited onto the glass substrate to increase the adhesion between the Ag nanorods and the glass substrate. For the GLAD process, the substrate was inclined at an angle of 85° between the incident incoming flux and normal of the substrate, and rotated at a speed of 5 rpm. The vertical Ag nanorods (500 nm) were formed on the substrate in the electron beam evaporation system under a vacuum condition of ~9.5 × 10^−7^ Torr at a deposition rate of ~5 Å/s. The length of the Ag nanorod was selected for maximizing the fluorescence enhancement factor based on our previous study [31]. Figure 2a,b show the top view and cross-sectional view, respectively, of the scanning electron microscopy (SEM) images of the fabricated MEF Ag nanorod substrate. Uniformly distributed high-density vertical Ag nanorods were formed on the substrate.

The mechanisms for the enhancement of fluorescence on vertical Ag nanorods MEF substrate can be categorized by the enhanced near-field, the increment in surface area, and the enhanced binding ability [31]. The most common reason for fluorescence enhancement on metallic nanostructure is the enhanced electromagnetic field generated by localized surface Plasmon resonance (LSPR). The surface area increment can enhance the fluorescence signal because more capture biomolecules can remain after the washing process due to the increased surface area. The enhanced binding ability, which is a structural characteristic of nanorods for holding the capture biomolecules, can increase the fluorescence signal in the same manner as the increased surface area. To examine the contribution of the enhanced near-field on the fluorescence enhancement factor, the fluorescence signals of the spotted streptavidin-conjugated Cy5 (Thermo-Fisher Scientific, Waltham, MA, USA) with 10 µg/mL concentration on the bare glass and Ag nanorods MEF substrates were measured just after the spotting process as shown in Figure S1 in the Supporting Information, because the fluorescence signals just after the spotting process were generated from the same number of fluorophore in both the glass and Ag nanorod MEF substrate. The enhancement factor of the Ag nanorod MEF substrate due to the enhanced near-field was ~ 5.8×. The total enhancement factor of the Ag nanorod MEF substrate due to all three mechanisms could be measured from the fluorescence signal measurement after the washing process as shown in Figure S2 of the Supporting Information. The total enhancement factor was ~210× and the additional ~36× enhancement might be due to the surface area increment and enhanced binding ability. These measured values using the protein were similar to the enhancement data using deoxyribonucleic acid (DNA) sample in previous research [31].

## 3. Development of the HSV-ID Chip

### 3.1. Fabrication and Analysis of the HSV-ID Chip

To develop the HSV-ID chip, semenogelin-2 antibody (sc-34722; Santa Cruz Biotechnology Inc., Dallas, TX, USA) and anti-17 beta estradiol antibody (ab20626; Abcam Co., Ltd., Cambridge, UK) were selected for the detection of semen and VF, respectively. Both antibodies were diluted with 200 μg/mL of 1× phosphate-buffered saline (PBS; Biosesang Inc., Seongnam, South Korea). Seven 10 nl spots of each antibody were spotted on a substrate using a nanodispenser device (PipeJet^®^; Bio-Fluidix GmbH Ltd., Freiburg, Germany). The substrate was stored at 4 °C for drying the spotted antibodies overnight. After the drying process, the substrate was immersed in a blocking solution (15% dry milk and 85% 1× PBS) and shaken using an orbital shaker (SHO-1D; Daihan Science Co., Ltd., Korea) at 55 rpm for 90 min. Finally, the substrate was washed with washing buffer (90% DI water, 10% 1× PBS, and 0.1% Tween^®^20) and dried by blowing filtered nitrogen. The fabricated HSV-ID chip was placed in a sealed Petri dish chamber and stored in a refrigerator at 4 °C.

For the detection experiment, the proteins in the detection sample (body fluid) were labeled with Dylight (Dylight™ 633; Thermo-Fisher Scientific, Waltham, MA, USA), which is a fluorescent dye with an excitation wavelength of 633 nm. The semen sample was diluted in 1× PBS buffer at a concentration of 6 mg/mL, and 50 μg of Dylight powder was mixed with the diluted detection sample at a volume of 500 μL. In the case of VF, a diluted sample with a concentration of 0.5 mg/mL was used. To remove unconjugated Dylight, a dialysis process was carried out using a membrane tube (Slide-A-Lyzer™ MINI Dialysis Unit; Thermo-Fisher Scientific, Waltham, MA, USA). The Dylight-mixed detection sample was injected into the membrane tube, and the membrane tube was placed in 1× PBS (1L). During the dialysis process, unconjugated Dylight was released through the membrane, and Dylight-conjugated proteins remained in the membrane tube. For the measurement experiment, the dialyzed detection sample was diluted with 500 ~ 10^6^ times in PBS.

The fabricated HSV-ID chip was attached to a 16-chamber aluminum jig, which could divide the slide glass of the microarray chip into 16 sections and provide a liquid chamber in each section. Therefore, a single slide glass of the HSV-ID chip could be used for the identification of 16 body fluid samples. The Dylight-conjugated detection sample (0.2 mL) was injected into a single chamber and incubated for antibody and biomarker binding at room temperature for 90 min (standard incubation time for a common antibody chip). After incubation, the chamber was washed with washing buffer 5 times and with DI water once to minimize nonspecific binding. For fluorescence analysis, the HSV-ID chip was detached from the Al chamber, and the fluorescence signal was measured using a microarray scanner (GenePix 4000B; Molecular Devices, Sunnyvale, CA, USA) with an excitation wavelength of 635 nm.

### 3.2. Effects of Chemical Modification of the MEF Substrate and Antibody

To improve the sensitivity of the HSV-ID chip, the amount of antibody probes remaining after the blocking and washing processes in the fabrication protocol should be increased. To increase the immobilization efficiency between the antibody and the GLAD-fabricated Ag nanorod substrate, an amine functional group was attached on the GLAD substrate, and a carboxyl functional group was attached to the antibody. The mechanism of covalent bonding between EDC-activated antibody and amine functionalized GLAD substrate was illustrated in Figure S3 in the Supporting Information. To attach an amine functional group on the GLAD substrate, the GLAD substrate was treated with O_2_ plasma for 10 s to activate the -OH bond. The O_2_ plasma-treated GLAD substrate was coated with (3-aminopropyl) triethoxysilane (Sigma-Aldrich Co, St. Louis, MO, USA) and incubated in a vacuum desiccator for 30 min at room temperature. The amine-functionalized GLAD substrate was subsequently washed 5 times with DI water to remove excess unbound (3-aminopropyl) triethoxysilane from the surface and dried with filtered nitrogen gas. For the confirmation of the amine functionalization on the GLAD substrate, X-ray photoelectron spectroscopy (XPS) analysis was carried out using the K-Alpha XPS system (Thermo-Fisher Scientific, Waltham, MA, USA) as shown in Figure S4 (Supporting Information). It clearly showed that the N atom was increased after amine functionalization on the vertical Ag nanorod MEF substrate. A well-known EDC activation method was used to make a covalent bonding of antibody with amine functionalized GLAD substrate. A reaction solution was prepared by mixing 1-ethyl-3-[3-dimethylaminopropyl] carbodiimide hydrochloride (EDC, Sigma-Aldrich Co, St. Louis, Mo, USA) and N-hydroxy-sulfo-succinimide (sulfo-NHS, Sigma-Aldrich Co, St. Louis, Mo, USA) in 0.1 M 2-(N-morpholino) ethane-sulfonic acid (MES) buffer (Biosesang Inc., Seongnam, South Korea) at a concentration of 0.4 mg/mL and 0.6 mg/mL, respectively. Then, 5 μL of antibody (200 μg/mL) was poured into 20 μL of the reaction solution and incubated for 15 min at 37 °C [32]. Finally, 50 μg/mL of carboxyl-functionalized antibody was obtained. During the incubation, the EDC reacted with a carboxylate (–COOH) of antibody and formed an amine-reactive O-acyl isourea intermediate. The intermediate can react with an amine functionalized on the Ag nanorod MEF substrate and make a stable amide bond. The addition of sulfo-NHS stabilized the amine-reactive intermediate by converting it to an amine-reactive sulfo-NSH ester [33]. Since the strong covalent amide bond between antibody and substrate can increase the amount of antibody probes after the blocking and washing process, more antibody–antigen reactions can happen in the identification process, which can increase the sensitivity.

**Figure 3:**
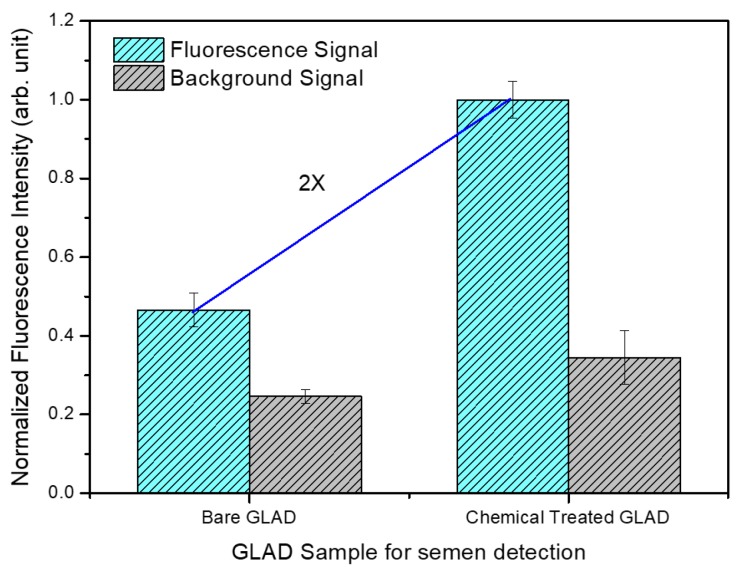
Comparison of the normalized fluorescence intensities in the semen detection experiment (12 ug/mL semen sample) using the bare GLAD HSV-ID chip and chemically-treated HSV-ID chip.

To examine the effectiveness of the chemical modification process, the EDC activated antibody (50 μg/mL) was spotted on the amine-functionalized GLAD substrate, and the same drying, blocking, and washing processes were carried out to obtain the chemically treated HSV-ID chip. The fluorescence signal from the chemically treated HSV-ID chip in the semen detection experiment was compared with that of the bare GLAD HSV-ID chip (concentration of initial spotted antibody = 200 μg/mL). A Dylight-conjugated human semen sample (12 μg/mL) was injected into the chamber and incubated for 90 min. After the washing process, the fluorescence signal was measured, as shown in Figure 3. In Figure 3, the fluorescence signal was the average intensity of the antibody spot location and the background signal was the average intensity of the non-spotting location. The fluorescence signal was generated from the specifically bound target protein and the background signal was generated from the non-specifically bound proteins. Although the initial concentration of the antibody spotting solution was reduced (around 4 times) in the chemically treated HSV-ID chip, the measured fluorescence signal of the chemically treated HSV-ID chip was around 2 times higher compared with that of the bare GLAD chip. The result demonstrated the effectiveness of the chemical treatment process, and the immobilization efficiency could be increased (8 times) using the proposed chemical treatment method. The slight increase in the background signal may be attributed to the nonspecific binding of unconjugated Dylight with the unblocked amine-functionalized GLAD substrate.

## 4. Evaluation of the HSV-ID Chip 

### 4.1. Identification of Semen and VF using the HSV-ID Chip

**Figure 4:**
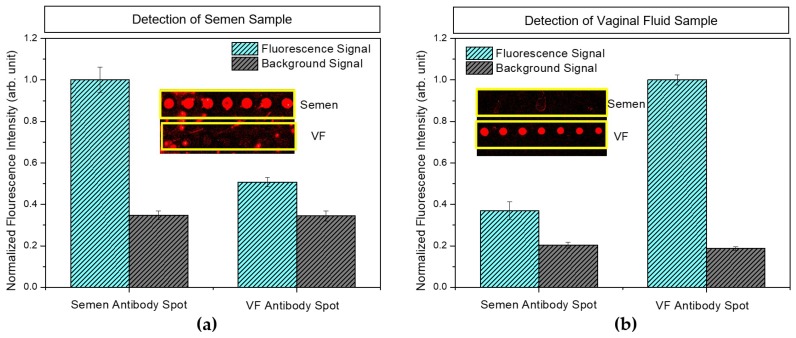
Measured fluorescence signal from the semen antibody spots and vaginal fluid (VF) antibody spots for (**a**) the 12 µg/mL semen sample and (**b**) the 1 µg/mL VF sample. Fluorescence images are inserted at the center of each graph (top: Semen antibody spots, bottom: VF antibody spots).

To examine the fabricated HSV-ID chip (with chemical treatment), the cross-reactivity of the HSV-ID chip was analyzed. A Dylight-conjugated semen sample (12 µg/mL) was injected into a chamber on the HSV-ID chip and incubated for 60 min. After the washing process, the fluorescence signal was measured as shown in Figure 4a. Fluorescence signals from the top seven spots (semen antibody spots) were observed with no cross-reactivity. Although some fluorescence signals were detected from the bottom seven spots (VF antibody spots), the signal intensity was similar to that of the auto-fluorescence signal of the VF antibody spots. For VF identification, a Dylight-conjugated VF sample (1 µg/mL) was injected into a chamber. After an incubation time of 60 min, the fluorescence signal was measured as shown in Figure 4b. The fluorescence image showed that a higher fluorescence signal was obtained from the VF antibody spots compared with the semen antibody spots. A small fluorescence signal was detected from the semen antibody spots, which may be attributed to the auto-fluorescence of the antibody itself. The background signal in Figure 4a,b may be attributed to the nonspecific binding of proteins on the substrate. In Figure 4, the intensity of the background signal in (b) was lower than in (a) because the concentration of the detection sample used in (b) (VF) was lower than in (a) (semen). This result clearly indicated that the developed HSV-ID chip could be used for the identification of semen and VF.

### 4.2. Effect of Incubation Time

**Figure 5:**
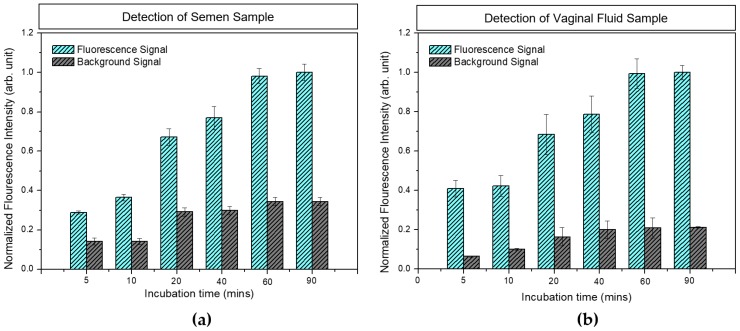
Effect of incubation time on the fluorescence signal for (**a**) the 12 µg/mL semen sample and (**b**) the 1 µg/mL vaginal fluid (VF) sample.

As the rapid identification of body fluids is desirable in crime investigations, the effect of incubation time on antibody-biomarker interaction during the detection process was analyzed. Samples of semen (12 μg/mL) and VF (1 μg/mL) were used for analyses. Figure 5 shows the measured fluorescence signal of (a) semen and (b) VF with different incubation times (5–90 min). With an increase in incubation time, the fluorescence intensity was increased, and saturation was observed after 60 min in both semen and VF samples. The background signal was also increased with increasing incubation time, possibly due to an increase in non-specific binding. Although an incubation time of 60 min was found to be optimum for obtaining the maximum fluorescence signal, the measured fluorescence signal following an incubation time of 5 min was also acceptable for the identification of 12 µg/mL semen and 1 µg/mL VF samples because the fluorescence signal was higher than the background signal. The results indicated that the fabricated HSV-ID chip could be used as a rapid identification tool for semen and VF.

### 4.3. Analysis of Detection Limit

**Figure 6:**
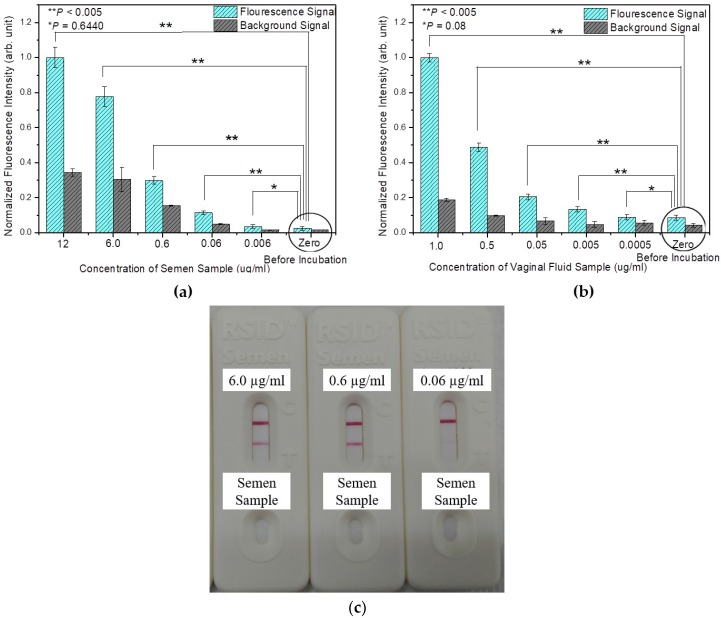
Limit of detection analysis results of the HSV-ID chip for (**a**) semen, (**b**) vaginal fluid, and (**c**) the limit of detection analysis results of the commercial RSID-Semen kit for semen.

In forensic analyses, the limit of detection (LOD) is important because of the limited sample amount at a crime site. We investigated the LOD of the fabricated HSV-ID chip for semen and VF and compared the measured LOD with that of the commercially available RSID-Semen kit for semen. The LOD for semen and VF was determined by applying samples with varying concentrations (12 µg/mL to 0.006 µg/mL for semen and 1.0 µg/mL to 0.0005 µg/mL for VF) to the HSV-ID chip. Figure 6 shows the measured fluorescence signals after an incubation time of 60 min for (a) semen and (b) VF samples with different concentrations. The fluorescence signal at 0 µg/mL was the signal from the HSV-ID chip before exposure to the detection sample. The fluorescence signal was decreased with decreasing semen and VF concentration. Although the fluorescence signal of semen at 0.006 µg/mL and VF at 0.005 µg/mL was not statistically different from the fluorescence signal at the 0 µg/mL, the signal at 0.06 µg/mL for semen and 0.005 µg/mL for VF were clearly distinguished statistically (*p* < 0.005). The LOD of the HSV-ID chip for identifying semen and VF was 0.06 µg/mL and 0.005 µg/mL, respectively.

The performance of the developed HSV-ID chip was compared with the commercially available RSID-Semen kit (RSID™ Semen; Independent Forensics, Lombard, IL, USA) under the same operating conditions. Figure 6c shows the detection of semen using the RSID-Semen kit at varying concentrations (6 µg/mL to 0.06 µg/mL). The semen concentration at 0.06 µg/mL was not detectable using the RSID-Semen kit; however, it was detectable using the HSV-ID chip. Overall, the LOD of the developed HSV-ID chip was around 10 times better than that of the commercially available RSID-Semen kit. As there is no commercially available VF identification chip, we could not compare the LOD of the HSV-ID chip for VF. 

## 5. Conclusions

An HSV-ID chip, a multiplex detection system for the identification of human semen and VF with a small amount of sample, was developed. Vertical Ag nanorods with a length of 500 nm were fabricated on Ni (10 nm) and Ag (50 nm) layers deposited on a glass substrate by GLAD. The surface of the Ag nanorods MEF substrate was modified with an amine functional group, and the detection antibody was modified with a carboxyl functional group to improve the immobilization efficiency, which further increased the fluorescence intensity (two times) and reduced the concentration of detection antibody (four times) compared with that of the bare GLAD HSV-ID chip. To examine the performance of the developed HSV-ID chip, the selectivity, incubation time, and LOD were determined. The fabricated HSV-ID chip demonstrated accurate selectivity with no cross-reactivity between the semen and VF samples. The fluorescence signal was increased with increasing incubation time, and it was saturated at 60 min. An incubation time of 5 min was also acceptable for the identification of both semen and VF samples. The LOD of the HSV-ID chip was 0.06 µg/mL for semen and 0.005 µg/mL for VF. The measured LOD of the HSV-ID chip for semen was 10 times better than that of the commercially available RSID-Semen kit. The validation study of the developed HSV-ID chip using various criminal samples and the extension of detectable body fluids including saliva, blood, and urine using the developed MEF substrate are the subjects of ongoing research.

## Supplementary Materials

The following are available online at http://www.mdpi.com/1424-8220/18/11/3874/s1, Figure S1: Comparison of measured fluorescence intensity of streptavidin-Cy5 spots just after spotting process (before washing) on bare glass substrate (reference), and vertical Ag nanorods MEF substrate. Figure S2: Comparison of measured fluorescence intensity of streptavidin-Cy5 spots after the washing process on bare glass substrate (reference), and vertical Ag nanorods MEF substrate. Figure S3: Schematic of the procedure to produce amine bond on the Ag nanorods MEF substrate and the activation of EDC group on the antibody for generating stable amide bond. Figure S4: The representative X-Ray Photoelectron Spectroscopy (XPS) spectra analysis of the surfaces of the Bare Ag MEF substrate and amine functionalized Ag MEF substrate.
