# Supplementary material for: Development of a Protein Microarray Chip with Enhanced Fluorescence for Identification of Semen and Vaginal Fluid
Source: Sensors (Basel). 2018 Nov 10;18(11):3874. doi: 10.3390/s18113874 (PMC6263525; doi:10.3390/s18113874)
Supplement: Supplementary file 1 [file sensors-18-03874-s001.pdf]

Supporting Information

Development of a Protein Microarray Chip with Enhanced Fluorescence for Identification of Semen and Vaginal Fluid

Naseem Abbas, Xun Lu, Mohsin Ali Badshah, Jung Bin In, Won Il Heo, Kui Young Park, Mi-Kyung Lee, Cho Hee Kim, Pilwon Kang, Woo-Jin Chang, Seok-min Kim and Seong Jun Seo

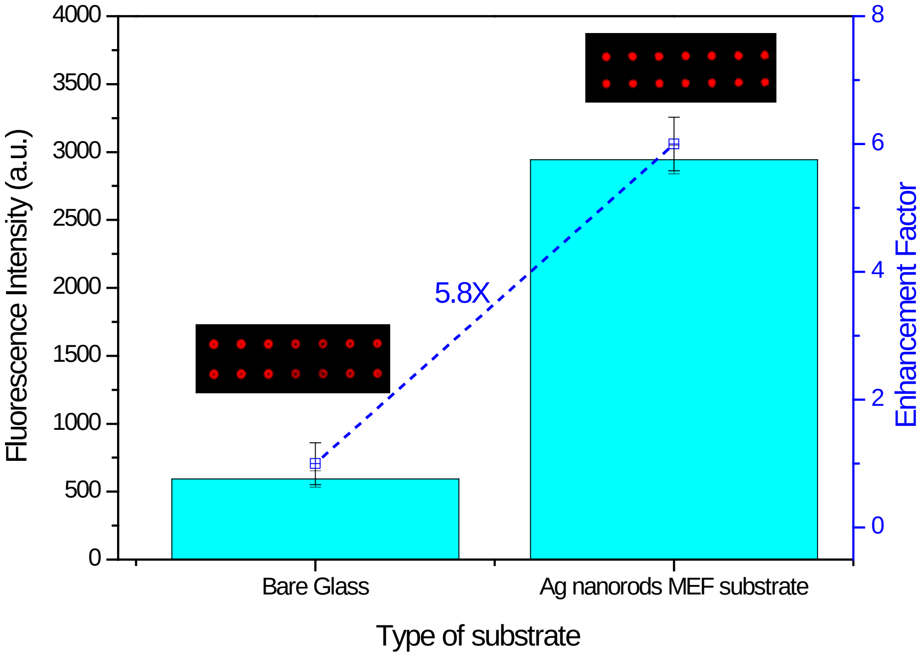

Figure S1. Comparison of measured fluorescence intensity of streptavidin-Cy5 spots just after spotting process (before washing) on bare glass substrate (reference), and vertical Ag nanorods MEF substrate.

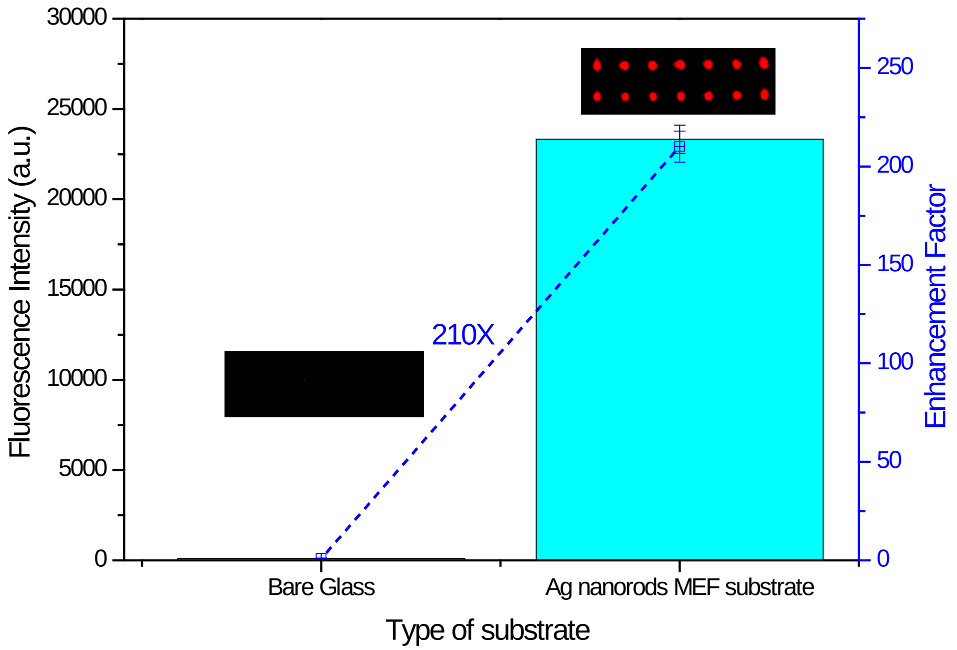

Figure S2. Comparison of measured fluorescence intensity of streptavidin-Cy5 spots after the washing process on bare glass substrate (reference), and vertical Ag nanorods MEF substrate.

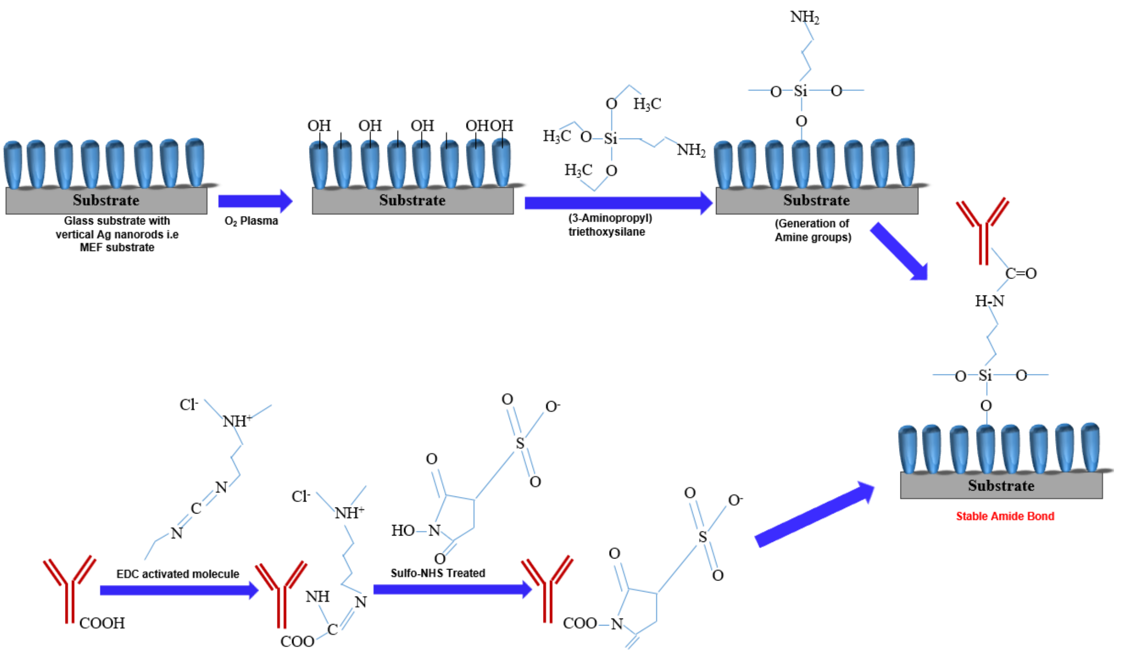

Figure S3. Schematic of the procedure to produce amine bond on the Ag nanorods MEF substrate and the activation of EDC group on the antibody for generating stable amide bond.

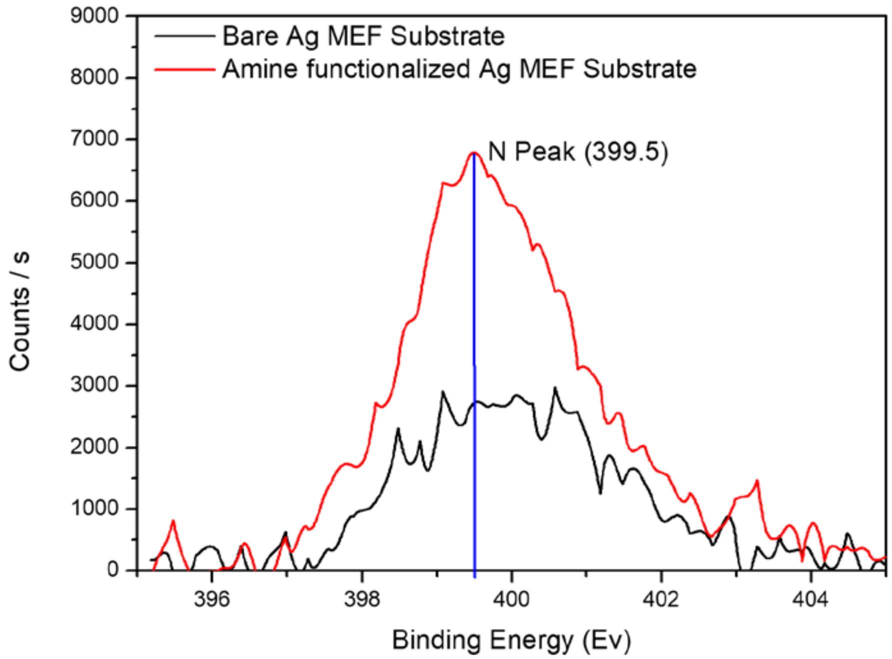

Figure S4. The representative X-Ray Photoelectron Spectroscopy (XPS) spectra analysis of the surfaces of the Bare Ag MEF substrate and amine functionalized Ag MEF substrate.
